# ﻿Two new species of *Chionosphaera* and *Kurtzmanomyces* (Chionosphaeraceae, Agaricostilbomycetes) isolated from China

**DOI:** 10.3897/mycokeys.125.167837

**Published:** 2025-11-14

**Authors:** Peng Wang, Chun-Yue Chai, Qiu-Hong Niu, Feng-Li Hui

**Affiliations:** 1 School of Life Science, Nanyang Normal University, Nanyang 473061, China Nanyang Normal University Nanyang China; 2 Research Center of Henan Provincial Agricultural Biomass Resource Engineering and Technology, Nanyang Normal University, Nanyang 473061, China Nanyang Normal University Nanyang China

**Keywords:** Basidiomycota, phylogenetic analysis, plant leaf, taxonomy, yeast

## Abstract

Within the family Chionosphaeraceae, only nine species of *Chionosphaera* and *Kurtzmanomyces* have been described to date. In this study, two strains of *Chionosphaera* and two strains of *Kurtzmanomyces* from China were examined using phylogenetic, physiological, and biochemical approaches. Phylogenetic analyses based on sequences of the internal transcribed spacer (ITS) region, the D1/D2 domain of the large subunit (LSU) rRNA gene, and the translation elongation factor 1-α gene (*tef1*α) were conducted to infer species relationships within the genera *Chionosphaera* and *Kurtzmanomyces*. As a result, two novel species are proposed: *Chionosphaera foliicola***sp. nov.** (holotype GDMCC 2.531^T^) and *Kurtzmanomyces guiyangensis***sp. nov.** (holotype GDMCC 2.499^T^). Descriptions and illustrations of both species are provided, together with comparisons to closely related taxa. This study expands the known diversity of Chionosphaeraceae in China and provides a basis for future taxonomic and ecological investigations.

## ﻿Introduction

Chionosphaeraceae (Agaricostilbomycetes, Agaricostilbales) is an ecologically significant family of basidiomycetous fungi with a broad environmental distribution. The family was originally established to accommodate teleomorphic taxa characterized by gasteroid basidia bearing basidiospores simultaneously on each basidium (Oberwinkler and Bandoni 1982; Bauer et al. 2006). Wang et al. (2015) revised the family to include five genera—*Ballistosporomyces*, *Chionosphaera*, *Cystobasidiopsis*, *Kurtzmanomyces*, and *Stilbum*—based on a phylogeny inferred from six gene loci. More recently, the genus *Boekhoutia* was added to the family (Li et al. 2020).

*Chionosphaera*, the type genus of Chionosphaeraceae, was first described by Cox (1976) and comprises species primarily associated with arboreal environments (Kyung and Kwon-Chung 2011). The genus was initially placed in the family Filobasidiaceae, but was later transferred to the newly established family Chionosphaeraceae based on molecular phylogenetic evidence (Oberwinkler and Bandoni 1982; Hibbett 2006). Wang et al. (2015) recognized six species within the genus: *C. apobasidialis*, *C. coppinsii*, *C. cuniculicola*, *C. erythrinae*, *C. lichenicola*, and *C. phylaciicola*. Subsequent molecular analyses led to the reclassification of *C. coppinsii* and *C. lichenicola* into the newly established genus *Crittendenia* within Pucciniomycotina, thereby excluding them from Chionosphaeraceae (Millanes et al. 2021). Currently, five species are accepted in the genus, including *C. pinicorticola*, a recently described species from South Korea (Lee et al. 2025). Among these, only *C. apobasidialis*, *C. cuniculicola*, and *C. pinicorticola* are known from living cultures, all exhibiting asexual yeast morphs (Kyung and Kwon-Chung 2011; Lee et al. 2025). The sexual morph occurs exclusively on natural substrates and forms minute, white, stipitate-capitate basidiocarps, typically bearing four to eight one-celled, hyaline, oblong to reniform or ellipsoid to cylindrical basidiospores (Kyung and Kwon-Chung 2011). In contrast, the asexual morph produces slightly mucoid, cream-colored colonies that reproduce by budding; some species may form pseudohyphae and/or true hyphae. Physiologically, *Chionosphaera* species do not ferment sugars or assimilate nitrate, usually react positively with diazonium blue B (DBB), and show weak or no urease activity (Lee et al. 2025).

*Kurtzmanomyces* was established by Yamada et al. (1988) to accommodate the asexual, stalk-producing yeast species *K. nectairii*, based on phenotypic characteristics. This taxonomic placement was later supported by molecular phylogenetic analyses (Fell et al. 2000). The genus was subsequently expanded to include three additional species: *K. tardus* and *K. insolitus* from Portugal (Giménez-Jurado et al. 1990; Sampaio et al. 1999), and *K. shapotouensis* from China (Zhang et al. 2013). Phylogenetic studies have shown that the sexual species *Mycogloea nipponica* is closely related to *Kurtzmanomyces* and produces a *Kurtzmanomyces*-like anamorph (Kirschner et al. 2003; Sampaio 2011). Species of *Kurtzmanomyces* are characterized by pale orange colonies and the production of blastoconidia at the tips of stalk-like conidiophores. Some species may also form ballistoconidia and septate hyphae (Yamada et al. 1988; Sampaio 2011). Physiologically, members of the genus generally lack fermentative ability, possess ubiquinone Q-10 as the major respiratory quinone, and can assimilate a variety of carbon sources, but not *myo*-inositol or D-glucuronate (Sampaio 2011).

During a survey of Chionosphaeraceae in China, four yeast isolates were obtained from plant leaf samples collected in Guizhou and Hainan Provinces. Molecular analyses based on the internal transcribed spacer (ITS) region, the D1/D2 domain of the large subunit (LSU) rRNA gene, and the translation elongation factor 1-α gene (*tef1*α), combined with physiological and biochemical data revealed that these isolates represent two novel species in the genera *Chionosphaera* and *Kurtzmanomyces*. The new taxa are described and illustrated below.

## ﻿Materials and methods

### ﻿Sample collection and yeast isolation

Plant leaf samples were collected from East Mountain Park (26°45'26"N, 106°21'31"E) in Guiyang City, Guizhou Province, and from Wuzhi Mountain (18°17'21"N, 109°40'55"E) in Wuzhishan City, Hainan Province, China. Out of the 19 collected plant leaf samples, strains NYNU 248111 and NYNU 248112 were isolated from two different leaf samples of *Symplocos adenophylla* collected in Wuzhi Mountain, while NYNU 23983 and NYNU 25716 were recovered from *Debregeasia orientalis* and *Glochidion* sp. in East Mountain Park and Wuzhi Mountain, respectively. These yeasts were isolated from the leaf surfaces using the washing and dilution method described by Jiang et al. (2024). Fresh leaves were cut into small pieces using sterile scissors, placed into sterile 10 mL centrifuge tubes, and suspended in sterile water containing 0.05 % (v/v) tween 80. The samples were shaken for 10 min, and the washing solution was serially diluted to 10^−2^. An aliquot of 200 μL from each dilution was spread onto yeast extract–malt extract (YM) agar medium (0.3 % yeast extract, 0.3 % malt extract, 0.5 % peptone, 1 % glucose, and 2 % agar) supplemented with 200 μg/mL chloramphenicol. The plates were then incubated at 20 °C until visible colonies developed. Colonies with distinct yeast morphologies were selected and purified by streaking onto fresh YM agar plates. Purified strains were suspended in 20 % (v/v) glycerol and stored at −80 °C for long-term preservation.

### ﻿Phenotypic studies

Morphological observations and physiological and biochemical tests were conducted following the methods described by Kurtzman et al. (2011). Carbon and nitrogen assimilation tests were performed twice in liquid media and monitored for up to 4 weeks. Nitrogen assimilation was tested using starved inocula. To induce sexual reproduction, single or double strains were mixed on corn meal agar (CMA: 2.5 % corn starch and 2 % agar), potato dextrose agar (PDA: 20 % potato infusion, 2 % glucose, and 2 % agar), and V8 agar (10 % V8 juice and 2 % agar) at 17 °C for 6 weeks, with observations made every two weeks (Li et al. 2020). Ballistoconidium formation was assessed by the inverted-plate method (do Carmo-Sousa and Phaff 1962) using CMA at 17 °C for two weeks. Temperature tolerance was determined by culturing on YM agar at 15, 20, 25, 30, 35, and 37 °C.

### ﻿DNA extraction, PCR amplification, sequencing

Genomic DNA was extracted from yeast cultures using the Ezup Column Yeast Genomic DNA Purification Kit (Sangon Biotech, Shanghai, China) following the manufacturer’s instructions. The ITS region, the D1/D2 domain of the LSU rRNA gene, and the partial *tef1*α gene were amplified with primers ITS1/ITS4 (White et al. 1990), NL1/NL4 (Kurtzman and Robnett 1998), and EF1-526F/EF1-1567R (Rehner and Buckley 2005), respectively. Each 25 μL PCR reaction consisted of 9.5 μL nuclease-free water, 12.5 μL of 2 × Taq PCR Master Mix with blue dye (Sangon Biotech, Shanghai, China), 1 μL genomic DNA template, and 1 μL of each primer. PCR amplification was conducted as described by Toome et al. (2013) for ITS and LSU. For *tef1*α, we used a touchdown PCR protocol as described (Wang et al. 2014). PCR products were analyzed by electrophoresis on 1 % agarose gels. Amplicons showing clear single bands were purified and sequenced by Sangon Biotech Co., Ltd. (Shanghai, China). Forward and reverse sequences were edited and assembled into consensus sequences using BioEdit v.7.1.3.0 (Hall 1999). All newly obtained sequences were deposited in GenBank (https://www.ncbi.nlm.nih.gov/genbank/).

### ﻿Sequence alignment and phylogenetic analyses

The obtained sequences were compared against the GenBank database using the BLASTn algorithm to identify closely related taxa (Altschul et al. 1997). For phylogenetic inference, all newly generated sequences were combined with reference sequences retrieved from GenBank (Table 1), following the taxon sampling frameworks proposed by Zhang et al. (2013) and Lee et al. (2025). The species of the genus *Stilbum* are not included in phylogenetic analyses because sequence data for type species of this genus are presently not available from public databases. *Naohidea sebacea* CBS 8477 and *Phyllozyma coprosmicola* CBS 7897 were designated as the outgroups. The ITS, LSU, and *tef1*α sequences were aligned separately using MAFFT v.7.110 (Katoh and Standley 2013) with the G-INS-i algorithm. Sequence alignments were visually inspected and manually refined in BioEdit v.7.1.3.0 (Hall 1999) to improve positional homology. The final ITS, LSU, and *tef1*α alignments were concatenated into a single dataset using PhyloSuite v.1.2.3 (Zhang et al. 2020) for subsequent phylogenetic analyses.

**Table 1.** Sequences used in phylogenetic analysis. Entries in bold are newly generated in this study.

Species	Strain no.	Locality	GenBank accession no.		
			ITS	LSU	*tef1α*
*Ballistosporomyces changbaiensis*	CBS 10124^T^	China	KP020105	KP020105	—
*Ballistosporomyces taupoensis*	CBS 7898^T^	Japan	NR_077093	NG_058698	KJ707901
*Ballistosporomyces xanthus*	CBS 7513^T^	Japan	NR_153596	NG_058697	KJ707902
*Boekhoutia sterigmata*	CGMCC 2.4539^T^	China	NR_174034	MK050371	—
*Chionosphaera apobasidialis*	CBS 7430^T^	USA	NR_073325	NG_042354	KJ707883
*Chionosphaera cuniculicola*	CBS 10063^T^	Germany	KJ778640	KJ708465	KJ707886
***Chionosphaera foliicola***	**NYNU 248111^T^**	**China**	**PV770167**	**PQ571723**	**PX046189**
***Chionosphaera foliicola***	**NYNU 248157**	**China**	**PV774782**	**PV774781**	**PX046190**
*Chionosphaera pinicorticola*	KACC 410729^T^	South Korea	PP702707	PP886164	—
*Cystobasidiopsis lactophilus*	CBS 7527^T^	Japan	NR_073299	NG_058726	KJ707889
*Cystobasidiopsis lophatheri*	CBS 11272^T^	Taiwan, China	NR_144767	NG_058719	KJ707880
*Cystobasidiopsis nirenbergiae*	TUB F580^T^	Germany	NR_158377	NG_058767	—
***Kurtzmanomyces guiyangensis***	**NYNU 23983^T^**	**China**	**OR961459**	**OR958742**	**PX046187**
***Kurtzmanomyces guiyangensis***	**NYNU 25716**	**China**	**PX046462**	**PX046461**	**PX046188**
*Kurtzmanomyces insolitus*	CBS 8377^T^	Portugal	NR_073322	NG_042355	KJ707893
*Kurtzmanomyces nectairei*	CBS 6405^T^	Japan	NR_073266	NG_042356	KJ707884
*Kurtzmanomyces shapotouensis*	CBS 12707^T^	China	NR_155216	NG_057975	—
*Kurtzmanomyces tardus*	CBS 7421^T^	Portugal	NR_073311	NG_042357	KJ707885
*Kurtzmanomyces* sp.	KBP Y-7120	Russia	OQ845641	OQ845641	—
‘*Rhodotorula*’ sp.	KBP Y-7115	Russia	OR195508	OR195508	—
*Kurtzmanomyces* sp.	KBP Y-6546	Russia	MW144956	MW144956	—
*Mycogloea nipponica*	CBS 11308	—	KJ778629	KJ708456	KJ707882
*Ruinenia bangxiensis*	CGMCC 2.3454^T^	China	NR_174775	MK050373	MK849035
*Ruinenia clavata*	CBS 9729^T^	China	NR_155708	NG_058390	KJ707894
*Ruinenia diospyri*	CBS 11271^T^	Taiwan, China	NR_144768	NG_064303	KJ707904
*Naohidea sebacea*	CBS 8477^T^	UK	NR_121324	NG_042442	KF706487
*Phyllozyma coprosmicola*	CBS 7897^T^	New Zealand	NR_073316	NG_058371	KJ707908

Strains marked with “T” are ex-type.

Maximum Likelihood (ML) and Bayesian Inference (BI) analyses were conducted using RAxML v.8.2.3 (Stamatakis 2014) and MrBayes v.3.2.7a (Ronquist et al. 2012), respectively. The ML analysis was performed under the GTR+G+I model, and branch support was evaluated with 1000 rapid bootstrap (BS) replicates. For BI, six Markov Chain Monte Carlo (MCMC) chains were run for 50 million generations under the best-fit substitution models selected using jModelTest (Posada 2008), with trees sampled every 1000 generations. The first 25 % of trees were discarded as burn-in, and the remaining trees were used to calculate Bayesian posterior probabilities (BPPs). Phylogenetic trees were visualized in FigTree v.1.4.3 (Andrew 2016), with support values indicated for nodes with BS ≥ 50% and BPPs ≥ 0.95.

## ﻿Results

### ﻿Molecular phylogeny

**Figure 1.**
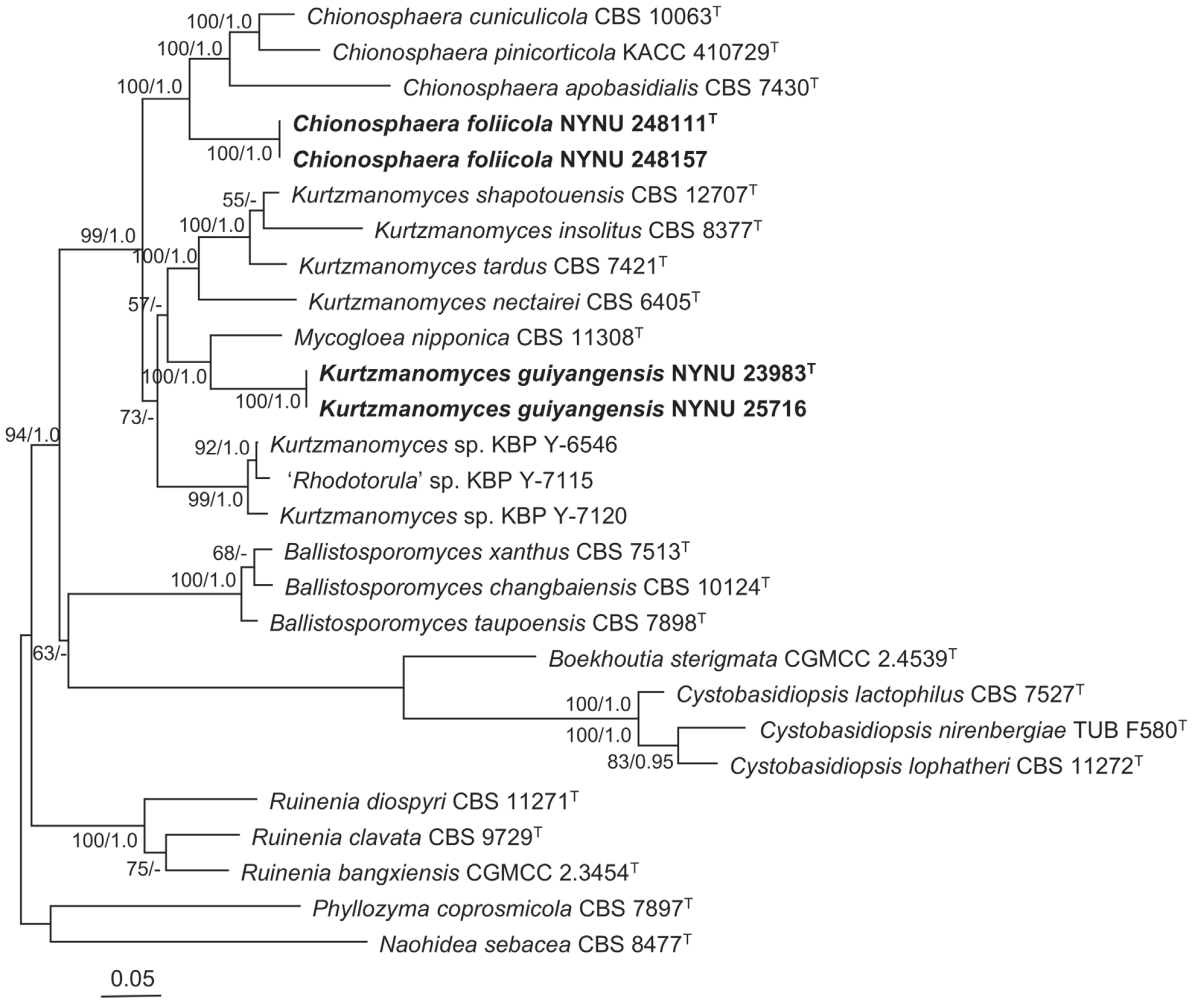
Phylogenetic tree of Chionosphaeraceae based on the ITS, LSU, and *tef1*α dataset. Bootstrap support (BS) ≥ 50% and Bayesian posterior probabilities (BPPs) ≥ 0.95 are shown. *Naohidea sebacea* CBS 8477 and *Phyllozyma coprosmicola* CBS 7897 were selected as the outgroups. New species are marked with bold.

In this study, 12 newly generated ITS, LSU, and *tef1*α sequences, together with 58 reference sequences retrieved from GenBank (Table 1), were included in the phylogenetic analyses. After removal of 656 ambiguously aligned regions (131 regions from ITS, 59 regions from LSU, and 466 regions from *tef1*α), the final concatenated alignment comprised 2332 base pairs, including 691 bp from ITS, 629 bp from LSU, and 1012 from *tef1*α. The ML and BI analyses yielded congruent topologies; therefore, only the ML tree is presented (Fig. 1). All Chionosphaeraceae taxa formed a strongly supported monophyletic clade (BS = 94 %, BPPs = 1.0). The four newly analyzed strains were grouped into two distinct lineages, which are here recognized as two putative novel species in the genera *Chionosphaera* and *Kurtzmanomyces*, respectively.

Strains NYNU 248111 and NYNU 248112, both isolated from *Symplocos adenophylla* collected in Wuzhi Mountain, exhibited identical ITS and LSU sequences, indicating that they are conspecific. Phylogenetic analyses placed these strains within the genus *Chionosphaera*, closely related to *C. cuniculicola*, *C. pinicorticola*, and *C. apobasidialis* (Fig. 1). However, the novel strains differed from these species by 37–68 nucleotide (nt) substitutions (~6.2–11.1 %) in the LSU region and by more than 58–80 nt differences (~9.6–13.3 %) in the ITS region. These levels of sequence divergence support their recognition as a distinct taxon. Although it cannot be ruled out that these strains represent the asexual morph of an already-known parasite with no sequence information available, it is meaningful to erect a new species in our opinion. Therefore, the name *Chionosphaera foliicola* sp. nov. is proposed for these two strains.

Strains NYNU 23983 and NYNU 25716 were isolated from *Debregeasia orientalis* and *Glochidion* sp. in East Mountain Park and Wuzhi Mountain, respectively. They were placed in the *Kurtzmanomyces* clade with an affinity to *M. nipponica* CBS 11308, a reference material of *M. nipponica*, based on phylogenetic analyses of the ITS, LSU, and *tef1*α sequences (Fig. 1). They differed from *M. nipponica* CBS 11308 by 36 nt substitutions (~5.3 %) in the LSU region and by more than 59 nt differences (~10.1 %) in the ITS region. These molecular differences, together with physiological and biochemical characteristics (see below), support its recognition as a distinct species. Due to the unavailability of *M. nipponica* type strain in reference culture collections (Sampaio 2011), we recommend keeping it classified as *M. nipponica* until additional molecular data are available. Therefore, we propose the name *Kurtzmanomyces guiyangensis* sp. nov. for this novel taxon.

### ﻿Taxonomy

#### ﻿ Chionosphaera foliicola

Taxon classificationFungiAgaricostilbomycetesChionosphaeraceae

C.Y. Chai & F.L. Hui sp. nov.

3DE5FD4B-C68A-5380-9EA0-9CB64D12D347

MB 859682

Fig. 2

##### Etymology.

The specific epithet *foliicola* refers to the ex-type strain isolated from a leaf.

**Figure 2.**
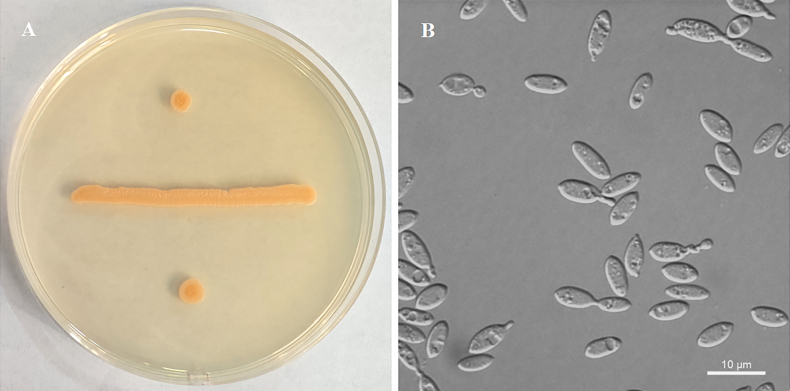
Morphology of *Chionosphaera foliicola* (NYNU 248111). A. Colony on YM agar after 7 days at 20 °C; B. Budding cells in YM broth after 3 days at 20 °C.

##### Typus.

**China** • Hainan Prov.: Wuzhishan City, Wuzhi Mountain, in the phylloplane of *Symplocos adenophylla*, August 2024, S.L. Lv, NYNU 248111 (holotype GDMCC 2.531^T^ preserved as a metabolically inactive state, culture ex-type PYCC 10141).

##### Description.

On YM agar after 7 days at 25 °C, the streak culture is pale orange tough, smooth and glossy with an entire margin. In YM broth after 3 days at 25 °C, cells are ellipsoidal to cylindrical (2.7–6.0 × 4.1–10.5 µm) and occur singly or in pairs. Budding is polar through minute stalks, normally one to two per cell. After a month in YM broth at 20 °C, a sediment is formed, but no ring and pellicle are observed. After 2 weeks in Dalmau plate culture on CMA at 20 °C, hyphae were observed. Sexual structures are not observed on PDA, CMA or V8 agar at 17 °C. Ballistoconidia are not produced. Sugar fermentation is absent. The following carbon sources are assimilated: glucose, inulin (delayed), sucrose, D-galactose (delayed), lactose (weak and delayed), trehalose, maltose, melezitose, methyl α-D-glucoside (delayed), cellobiose, salicin (weak), L-sorbose (weak and delayed), L-rhamnose (weak and delayed), D-xylose (weak and delayed), L-arabinose (weak and delayed), D-arabinose (weak), 5-keto-D-gluconate, ethanol (weak), glycerol (weak and delayed), ribitol (weak), galactitol, D-mannitol, D-glucitol (weak and delayed), DL-lactate (weak and delayed), succinate, citrate (weak and delayed), and d-glucono-1,5-lactone. Raffinose, melibiose, d-ribose, methanol, erythritol, *myo*-inositol, D-glucosamine, N-acetyl-D-glucosamine, 2-keto-d-gluconate, and D-glucuronate are not assimilated. L-Lysine is assimilated as sole nitrogen sources. Nitrate, nitrite, ethylamine, and cadaverine are not assimilated. Growth is observed at 30 °C, but not at 35 °C. Growth in the presence of 0.01 % cycloheximide, 10 % NaCl plus 5 % glucose as well as 1% acetic acid is negative. Growth in the vitamin-free medium is positive. Growth on 50 % (w/w) glucose-yeast extract agar is negative. There is no hydrolysis of urea and no starch formation. The DBB reaction is positive.

##### Additional strain examined.

**China** • Hainan Prov.: Wuzhishan City, Wuzhi Mountain, in the phylloplane of *Symplocos adenophylla*, August 2024, S.L. Lv, NYNU 248157.

##### GenBank accession numbers.

Holotype GDMCC 2.531^T^ (ITS: PV770167, LSU: PQ571723; *tef1*α: PX046189); additional strain NYNU 248157 (ITS: PV774782, LSU: PV774781; *tef1*α: PX046190).

##### Note.

Physiologically, *C. foliicola* sp. nov. differs from the closely related species, *C. apobasidialis*, *C. cuniculicola*, and *C. pinicorticola*, in its ability to assimilate sucrose and lactose (Table 2).

**Table 2.** Physiological and biochemical characteristics of the new species and their closely related taxa.

Characteristic	1	2^*^	3^*^	4^*^	5	6^*^	7^*^	8^*^	9^*^	10^*^
Fermentation of										
Glucose	–	–	n	n	–	–	–	–	+	–
Galactose	–	n	n	n	–	–	–	–	+	–
Sucrose	–	n	n	n	–	–	–	–	+	–
Raffinose	–	n	n	n	–	–	–	–	+	–
Trehalose	–	n	n	n	–	–	–	–	+	–
Assimilation of										
Inulin	d	s	+	–	+	–	–	–	+	–
Sucrose	+	–	–	–	+	+	+	–	+	+
Raffinose	–	–	–	–	–	–	+	–	+	+
Galactose	d	w	w	–	–	–	–	–	+	+
Lactose	d, w	–	–	–	–	–	+	–	–	–
Trehalose	+	v	+	+	+	–	+	+	+	+
Maltose	+	–	–	s	–	–	–	–	–	+
Melezitose	+	–	+	–	+	–	+	–	+	+
Cellobiose	+	–	w	–	–	–	+	–	–	+
Salicin	w	v	–	–	+	–	S	–	–	s
L-Sorbose	d, w	+	w	v	–	–	–	–	+	–
D-Xylose	d, w	–	–	v	–	–	+	–	+	s
L-Arabinose	d, w	s	w	v	+	–	+	–	+	–
D-Arabinose	w	+	w	–	+	–	+	–	+	–
D-Ribose	–	–	–	–	+	–	S	–	+	s
Glycerol	d, w	+	+	–	–	–	S	–	–	s
Erythritol	–	–	–	–	–	–	S	–	–	+
Ribitol	w	v	+	s	+	–	S	+	+	+
D-Glucitol	d, w	s	+	v	+	w, s	–	+	n	–
Growth tests										
0.1 % Cycloheximide	–	n	n	n	–	–	+	–	–	–
Vitamin-free medium	w	s, w	n	n	–	–	–	–	+	–
Growth at 30 °C	+	+	n	w, –	–	+	+	–	+	+

Species: 1, *C. foliicola* nov.; 2, *C. cuniculicola*; 3, *C. pinicorticola*; 4, *C. apobasidialis*; 5, *K. guiyangensis* sp. nov.; 6, *K. tardus*; 7, *K. insolitus*; 8, *K. nectairei*; 9, *K. shapotouensis*; 10, *M. nipponica*. ^*^Data for reference species were taken from Kyung and Kwon-Chung (2011), Zhang et al. (2013), Lee et al. (2025), and Sampaio (2011). +, positive; –, negative; d, delayed positive (growth after > 7 days); s, slow positive (growth after > 2 weeks); v, variable (some strains are positive, others negative); w, weakly positive (< 25% of control growth); n, not available.

#### ﻿ Kurtzmanomyces guiyangensis

Taxon classificationFungiAgaricostilbomycetesChionosphaeraceae

C.Y. Chai & F.L. Hui sp. nov.

583A6DD5-B967-5C6D-BABF-99BDF35E3D08

MB 859684

Fig. 3

##### Etymology.

The specific epithet *guiyangensis* refers to the geographic origin of the ex-type strain, Guiyang City, Guizhou Province.

**Figure 3.**
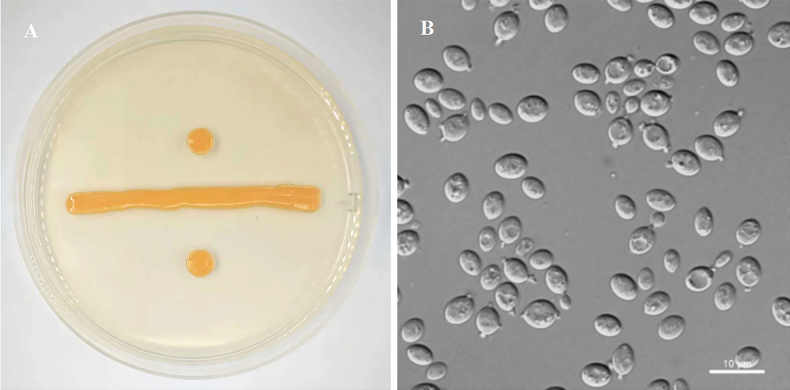
Morphology of *Kurtzmanomyces guiyangensis* (NYNU 23983). A. Colony on YM agar after 7 days at 20 °C; B. Budding cells in YM broth after 3 days at 20 °C.

##### Typus.

**China** • Guizhou Prov.: Guiyang City, East Mountain Park, in the phylloplane of *Debregeasia orientalis*, August 2023, D. Lu, NYNU 23983 (holotype GDMCC 2.499^T^ preserved as a metabolically inactive state, culture ex-type PYCC 9992).

##### Description.

On YM agar after 7 days at 20 °C, streak cultures are pale orange, smooth and butyrous, and the margin is entire. In YM broth after 3 days at 20 °C, cells are spherical to ovoid (3.4–4.7 × 4.5–6.8 µm) and occur singly or in pairs. The conidiogenous stalks, normally one to three per cell, are short, 0.4–1.0 µm. After a month in YM broth at 17 °C, a sediment and a ring are formed, but no pellicle is observed. After 7 days in Dalmau plate culture on CMA at 17 °C, pseudohyphae are absent. Sexual structures are not observed on PDA, CMA or V8 agar at 17 °C. Ballistoconidia are not produced. Glucose fermentation is absent. The following carbon sources are assimilated: glucose, inulin, sucrose, trehalose, melezitose, salicin, L-arabinose, D-arabinose, 5-keto-D-gluconate, D-ribose, ethanol, ribitol, galactitol, D-mannitol, D-glucitol, and succinate. Raffinose, melibiose, D-galactose, lactose, maltose, methyl α-D-glucoside, cellobiose, L-sorbose, L-rhamnose, D-xylose, methanol, glycerol, erythritol, *myo*-inositol, DL-lactate, citrate, D-glucuronate, D-glucosamine, N-acetyl-D-glucosamine, 2-keto-D-gluconate, and D-glucono-1,5-lactone are not assimilated. Nitrate and nitrite are weakly assimilated as sole nitrogen sources. Ethylamine, l-lysine, and cadaverine are not assimilated. Growth is observed at 25 °C, but not at 30 °C. Growth in the presence of 0.01 % cycloheximide, 10 % NaCl plus 5 % glucose as well as 1 % acetic acid is negative. Growth in the vitamin-free medium is absent. Growth on 50 % (w/w) glucose-yeast extract agar is positive. Starch-like compounds are not synthesized. Diazonium blue B reactions is positive.

##### Additional strain examined.

**China** • Hainan Prov.: Wuzhishan City, Wuzhi Mountain, in the phylloplane of *Glochidion* sp., July 2025, S.L. Lv, NYNU 25716.

##### GenBank accession numbers.

Holotype GDMCC 2.499^T^ (ITS: OR961459, LSU: OR958742, *tef1*α: PX046187); additional strain NYNU 25716 (ITS: PX046462, LSU: PX046461; *tef1*α: PX046188).

##### Note.

Physiologically, *K. guiyangensis* sp. nov. differs from the closely related species *M. nipponica* in its ability to assimilate inulin, L-arabinose, D-arabinose, and D-glucitol and its inability to assimilate raffinose, galactose, maltose, and glycerol. Furthermore, *M. nipponica* is able to grow at 30 °C, whereas *K. guiyangensis* sp. nov. does not exhibit growth at this temperature. A comparative summary of the distinguishing physiological characteristics between *K. guiyangensis* sp. nov. and other *Kurtzmanomyces* species is presented in Table 2.

## ﻿Discussion

In this study, four strains belonging to the family Chionosphaeraceae were isolated during an investigation of leaf-inhabiting fungi from different regions of China. Based on a polyphasic approach combining physiological, biochemical, and molecular data, two novel species were proposed in the genera *Chionosphaera* and *Kurtzmanomyces*. The phylogenetic analyses presented here are consistent with previous studies (Zhang et al. 2013; Lee et al. 2025) and contribute additional insights into the taxonomy and phylogenetic relationships within Chionosphaeraceae.

*Chionosphaera* species predominantly inhabit tree bark and other natural substrates. For instance, *C. apobasidialis* was isolated from decayed bark of *Quercus macrocarpa*, *C. cuniculicola* from beetles in coniferous forests, *C. erythrinae* from leaves of *Erythrina tomentosa*, and *C. pinicorticola* from pine bark, collectively indicating a preference for arboreal habitats (Cox 1976; Kirschner et al. 2001; Kirschner and Chen 2008; Lee et al. 2025). In line with this ecological tendency, the newly described species *C. foliicola* sp. nov. was isolated from the phylloplane of *Symplocos adenophylla*. Although its complete ecological role in situ remains to be fully understood, its occurrence in a similar arboreal environment suggests that it shares ecological preferences with its congeners.

To date, four asexual species have been described in the genus *Kurtzmanomyces*. These species are rarely encountered in nature, with most known only from a single isolate. *K. insolitus* was isolated from the basidiocarp of an unidentified *Exidiopsis* species in Portugal (Sampaio et al. 1999), *K. nectairei* from cheese in France (Rodrigues de Miranda, 1975), *K. shapotouensis* from soil crusts in China (Zhang et al. 2013), and *K. tardus* from contaminated demineralized water in Portugal (Giménez-Jurado et al. 1990). In addition, *Mycogloea nipponica*, which possesses a *Kurtzmanomyces*-like anamorph, appears to be a mycoparasite on ascomycetes and has been reported from Japan and Taiwan (Bandoni 1998; Kirschner et al. 2003). Furthermore, at least three unpublished or misidentified yeasts —*Kurtzmanomyces* sp. KBP Y-7120, ‘*Rhodotorula*’ sp. KBP Y-7115, and *Kurtzmanomyces* sp. KBP Y-6546 —may represent additional novel species of the genus (Fig. 1). All three were isolated from the lichen *Cladonia stellaris* in Russia. In this study, *K. guiyangensis* sp. nov. was recovered from the leaf surfaces of *Debregeasia orientalis* and *Glochidion* sp. in different regions of China. This distinct ecological origin suggests that *K. guizhouensis* occupies a different ecological niche compared to previously known members of the genus *Kurtzmanomyces*. This expands the known ecological range of *Kurtzmanomyces* and underscores the importance of exploring diverse habitats for uncovering yeast diversity.

## Supplementary Material

XML Treatment for Chionosphaera foliicola

XML Treatment for Kurtzmanomyces guiyangensis

## ﻿Additional information

### Use of AI

No use of AI was reported.

### Author ORCIDs

Peng Wang https://orcid.org/0009-0001-8289-9640

Chun-Yue Chai https://orcid.org/0000-0001-7753-6223

Qiu-Hong Niu https://orcid.org/0000-0003-1695-7117

Feng-Li Hui https://orcid.org/0000-0001-7928-3055

### Data availability

All of the data that support the findings of this study are available in the main text or Supplementary Information.

## Supplementary materials

Supplementary material 1 A dataset of ITS, LSU, and *tef1α* for Fig. 1 Data type fasta https://binary.pensoft.net/file/1466018This dataset is made available under the Open Database License (http://opendatacommons.org/licenses/odbl/1.0/). The Open Database License (ODbL) is a license agreement intended to allow users to freely share, modify, and use this Dataset while maintaining this same freedom for others, provided that the original source and author(s) are credited.Peng Wang, Chun-Yue Chai, Qiu-Hong Niu, Feng-Li Hui
